# 17**β**-Estradiol Attenuates Poststroke Depression and Increases Neurogenesis in Female Ovariectomized Rats

**DOI:** 10.1155/2013/392434

**Published:** 2013-11-07

**Authors:** Yifan Cheng, Qiaoer Su, Bei Shao, Jianhua Cheng, Hong Wang, Liuqing Wang, Zhenzhen Lin, Linhui Ruan, Qichuan ZhuGe, Kunlin Jin

**Affiliations:** ^1^Zhejiang Provincial Key Laboratory of Aging and Neurological Disorder Research, First Affiliated Hospital, Wenzhou Medical University, Wenzhou 35000, China; ^2^Department of Neurology, First Affiliated Hospital, Wenzhou Medical University, Wenzhou 325000, China; ^3^Department of Pharmacology and Neuroscience, Institute for Aging and Alzheimer's Disease Research, University of North Texas Health Science Center at Fort Worth, 3500 Camp Bowie Boulevard, Fort Worth, TX 76107, USA

## Abstract

Studies have linked neurogenesis to the beneficial actions of specific antidepressants. However, whether 17**β**-estradiol (E_2_), an antidepressant, can ameliorate poststroke depression (PSD) and whether E_2_-mediated improvement of PSD is associated with neurogenesis are largely unexplored. In the present study, we found that depressive-like behaviors were observed at the first week after focal ischemic stroke in female ovariectomized (OVX) rats, as measured by sucrose preference and open field test, suggesting that focal cerebral ischemia could induce PSD. Three weeks after middle cerebral artery occlusion (MCAO), rats were treated with E_2_ for consecutive 14 days. We found that E_2_-treated rats had significantly improving ischemia-induced depression-like behaviors in the forced-swimming test and sucrose preference test, compared to vehicle-treated group. In addition, we also found that BrdU- and doublecortin (DCX)-positive cells in the dentate gyrus of the hippocampus and the subventricular zone (SVZ) were significantly increased in ischemic rats after E_2_ treatment, compared to vehicle-treated group. Our data suggest that focal cerebral ischemia can induce PSD, and E_2_ can ameliorate PSD. In addition, newborn neurons in the hippocampus may play an important role in E_2_-mediated antidepressant like effect after ischemic stroke.

## 1. Introduction

Poststroke depression (PSD) is the most frequent and important neuropsychiatric consequence of stroke, which occurs about 33% of all stroke survivors [1, 2]. Compared to stroke patients without depression, patients with PSD were found to be associated with increases in physical disability, cognitive impairment, mortality, and risk of falling, as well as with worsened rehabilitation outcome [3]. Although there have been abundant papers focused on PSD regarding epidemiological features and impact of PSD both on functional outcome, the evidence for effective treatments for PSD remains largely under developed [2].

Estrogens are a group of steroid compounds that functions in the reproductive system, as well as in nonreproductive tissue such as the skeletal and cardiovascular systems. Estrogen treatment to ovariectomized (OXV) female rats significantly reduced the infarct volume [4] and improved sensorimotor dysfunction after focal ischemia [5–7]. In addition to its action on neuroprotection, estrogen is also demonstrated to be beneficial for improving depressive mood in women with reproductive-related mood disorders, including postpartum depression [8] and perimenopausal depressive disorders [9]. Animal studies suggest that estrogen administration can reduce immobility time in the forced swimming test, a paradigm used to test the efficacy of antidepressants and immobility means of depression. Interestingly, recent studies also reveal that estrogen may play a significant role in modulating adult neurogenesis [10, 11]. Administration of 17*β*-estradiol after ischemic stroke profoundly enhanced neurogenesis by increasing the number of newborn neurons in the subventricular zone (SVZ) and facilitating migration of newborn neurons to ischemic regions [12].

Neurogenesis is a continuous process of the generation of new neurons, which occurs throughout adulthood primarily in the dentate gyrus (DG) of the hippocampus and the SVZ. Current evidence indicate that there is a link between adult hippocampal neurogenesis and depression [13, 14]. Several risk factors for clinical depression, such as chronic stress [15], alcohol abuse [16], infection [17], and neurodegenerative disorders [18], also suppress neurogenesis in the adult hippocampus. Although it is controversial whether impaired neurogenesis is sufficient to cause depressive phenotype, the role of neurogenesis in mediating therapeutic efficacy of antidepressants in depression is recognized [19]. It is well accepted that the behavioral effects of antidepressants is partly mediated by the stimulation of hippocampal neurogenesis. Most antidepressants that confer antidepressant-like behavioral effects induce adult hippocampal neurogenesis by upregulating molecular pathways involving monoamine release [20], activation of serotonin 1A receptor [19], and neurotrophic factor expression [21]. These findings led us to investigate whether estrogen can improve the PSD symptom and its underlying mechanisms.

In this study, we examined the therapeutic potential of 17*β*-estradiol (E_2_) in poststroke depression. We found that E_2_ treatment reduced depressive-like behavior and significantly promoted neurogenesis in the DG of the hippocampus and the SVZ after focal cerebral ischemia. Our data suggest that estrogen-induced neurogenesis may play a critical role in antidepressant therapy in PSD.

## 2. Material and Methods

### 2.1. Experimental Animals

Female Sprague-Dawley (SD) rats (250–300 g) were housed four per cage under conditions of constant temperature (23 ± 1°C) and humidity (50%) in a 12:12 hr light-dark cycle with ad libitum access to food and water. Four groups of ovariectomized female SD rats were used in our experiment: (1) sham-operated rats treated with vehicle (sham + vehicle); (2) sham-operated rats treated with 17*β*-estradiol (sham + E_2_); (3) left middle cerebral artery occlusion (MCAO) rats treated with vehicle (MCAO + vehicle); (4) MCAO rats treated with 17*β*-estradiol (MCAO + E_2_). All procedures were conducted in accordance with the Guidelines of the Chinese Council on Animal Care and approved beforehand by the Institutional Animal Care and Use Committee of Wenzhou Medical University.

### 2.2. Ovariectomy and 17*β*-Estradiol Treatment

Female SD rats were bilaterally OVX under chloral hydrate anesthesia and aseptic conditions. Briefly, a single midline incision was made in the low abdominal area to expose the ovary; oviducts were bilaterally ligated and ovaries removed. After suturing their muscles and skin, the animals were returned to their home cages to recover for one week. The hormone therapy began 3 weeks after surgery. 17*β*-Estradiol (E_2_; sigma; 10 **μ**g) was dissolved in 0.1 mL of soybean oil and administered subcutaneously for consecutive 14 days.

### 2.3. Behavioral Testing

#### 2.3.1. Sucrose Preference Test (SPT)

The SPT was performed as described by Benelli et al. [22]. Briefly, before testing, rats were exposed to a solution of 1% sucrose for 24 hr without any food and get habituated to consuming sucrose solution, during the subsequent 24 hr, one bottle contained the sucrose solution, the other contained tap water. After 23 hr of deprivation of food and water, each rat was provided with two identical bottles, one with 1% sucrose solution and another with tap water. The amount of water and 1% sucrose solution intake was recorded after a 1 hr test. Data were expressed as percentage of 1% sucrose consumption from total consumption.

#### 2.3.2. Open Field Test (OFT)

The OFT was performed to evaluate general locomotor and rearing activity of the rats as described by Wang et al. [23]. The apparatus consisted of a dark varnished wooden box (100 × 100 × 40 cm^3^) with the floor divided into 25 equal squares. Rats were gently placed on the center square and left to explore the floor for 3 min. The measurement parameters of this test include locomotor activity registered as the number of times the animal crosses squares and the rearing activity, which was registered as the number of times the animal stands upright on its hind legs. Both locomotor activity and rearing activity were manually recorded over a 3 min period by trained observers who were blind to the experimental design.

#### 2.3.3. Forced Swimming Test (FST)

The modified FST was performed essentially as described by Detke and his colleague [24]. On the first day, the rats were individually placed in a glass cylinder (45 cm height × 18 cm diameter) containing 30 cm of water at 23–25°C for 15 min. The rats were then removed from the cylinder, dried with tissue paper, and returned to their home cage. On the second day, the rats were placed in the cylinder for 5 min again, and behaviors were scored by observers unaware of experimental groups. Three different behaviors were scored: (1) climbing—presenting active movements with the forepaws in and out of the water, usually directed against the wall of tank; (2) swimming—showing active movements using forepaws and hindpaws within the tank that mimicked swimming motions; (3) Immobility—floating in the water without struggling and doing only those movements necessary to keep the head above the water.

Sucrose preference test and open field test were performed weekly to assess endogenous depressive-like behavior after focal ischemia for three weeks. Likewise, sucrose preference test and forced swimming test were performed weekly to assess behavioral changes after E_2_ administration. As the same subjects were used for the behavioral tests, we performed sucrose preference test, and animals were allowed to recover for a day.

### 2.4. Transient Focal Cerebral Ischemia

Female SD rats were anesthetized with 8% chloral hydrate. The rectal temperature was maintained at 37.0–37.5°C with a heating blanket throughout the operation. Transient focal cerebral ischemia was induced by occlusion of the left middle cerebral artery (MCA) as described previously [25]. After a midline incision in the neck, the left external carotid artery was ligated and dissected distally, and the left internal carotid was isolated from the vagus nerve. The embolus, made up of nylon suture with rounded tip, was inserted into the left internal carotid through a small incision into the external carotid artery and was gently advanced 20-21 mm past the carotid bifurcation to occlude the left MCA. The embolus was left in place for 90 min and then removed to allow reperfusion. Sham-operated animals were treated identically except that the MCA was not occluded after the neck incisions.

### 2.5. Measurement of Infarct Volume

The rat brains were removed, and 50 **μ**m coronal sections were cut and stained with cresyl violet. Contralateral and ipsilateral hemisphere areas were measured by a blinded observer using the NIH Image program, and areas were multiplied by the distance between sections to obtain the respective volumes. Volume loss (mm^3^) was calculated as a percentage of the volume of the structures in the control hemispheres according the following formula: (100 × (*V*_*C*_ − *V*_*L*_)/*V*_*C*_ (*V*_*C*_ = control hemisphere volume, *V*_*L*_ = lesioned hemisphere volume)), as described previously [25].

### 2.6. BrdU Administration

BrdU (50 mg kg^−1^ in saline) was administered intraperitoneally twice daily for 3 consecutive days before rats were euthanized. The rats were perfused transcardially with 4% PFA in PBS, and brains were postfixed overnight and embedded in paraffin.

### 2.7. Immunohistochemistry

Immunohistochemistry (5-6 animals per group) was performed as described previously [25]. Primary antibodies were mouse monoclonal anti-BrdU (2 **μ**g/mL; Roche) and affinity-purified goat anti-DCX (1 : 200; Santa Cruz Biotechnology); secondary antibodies were biotinylated donkey anti-goat or biotinylated horse anti-mouse IgG (both 1 : 200; Santa Cruz Biotechnology). Sections were examined with a Nikon E800 epifluorescence microscope. Controls included omitting the primary and secondary antibodies.

### 2.8. Dual-Label Immunohistochemistry

Dual-label immunohistochemistry (5-6 animals per group) was performed as described elsewhere [26]. Primary antibodies were those listed above; secondary antibodies were Alexa Fluor 488-, 594-, or 647-conjugated donkey anti-mouse or anti-goat IgG (1 : 200–500; Molecular Probes). Fluorescence signals were detected using an LSM 510 NLO Confocal Scanning System mounted on an Axiovert 200 inverted microscope (Carl Zeiss) equipped with a two-photon Chameleon laser (Coherent), and images were acquired using LSM 510 Imaging Software (Carl Zeiss). Two- or three-color images were scanned using Ar, 543 HeNe, 633 HeNe, and Chameleon lasers. Selected images were viewed at high magnification. Controls included omitting either the primary or secondary antibody or preabsorbing the primary antibody.

### 2.9. Cell Counting

BrdU- and DCX-positive cells in SVZ and DG were counted in five to seven 50 **μ**m coronal sections per animal (*n* = 6 per group), spaced 200 **μ**m apart, by an observer blind to the experimental condition using a Zeiss microscope in bright field mode and a 40X objective. Confocal microscopy was used to count double-labeled cells. In SVZ, DCX- or BrdU-labeled cells were counted along the lateral walls of the lateral ventricles for a total of five to six sections per rat. For the DG, all DCX- or BrdU-labeled cells within two cell diameters from the inner edge of the granule cell layer (GCL) of the DG were included in the analysis. Results were expressed as the average number of BrdU- and DCX-positive cells in SVZ and DG per section.

### 2.10. Statistical Analysis

All quantitative data were expressed as mean ± SEM. Behavioral data were analyzed by a repeated measurement analysis of variance (ANOVA). The neurogenesis cell count data was analyzed by one way ANOVA followed by LSD *post hoc* test. *P* values < 0.05 were considered statistically significant.

## 3. Results

### 3.1. Depressive-Like Behaviors Were Observed in Poststroke Rats

**Figure 1:**
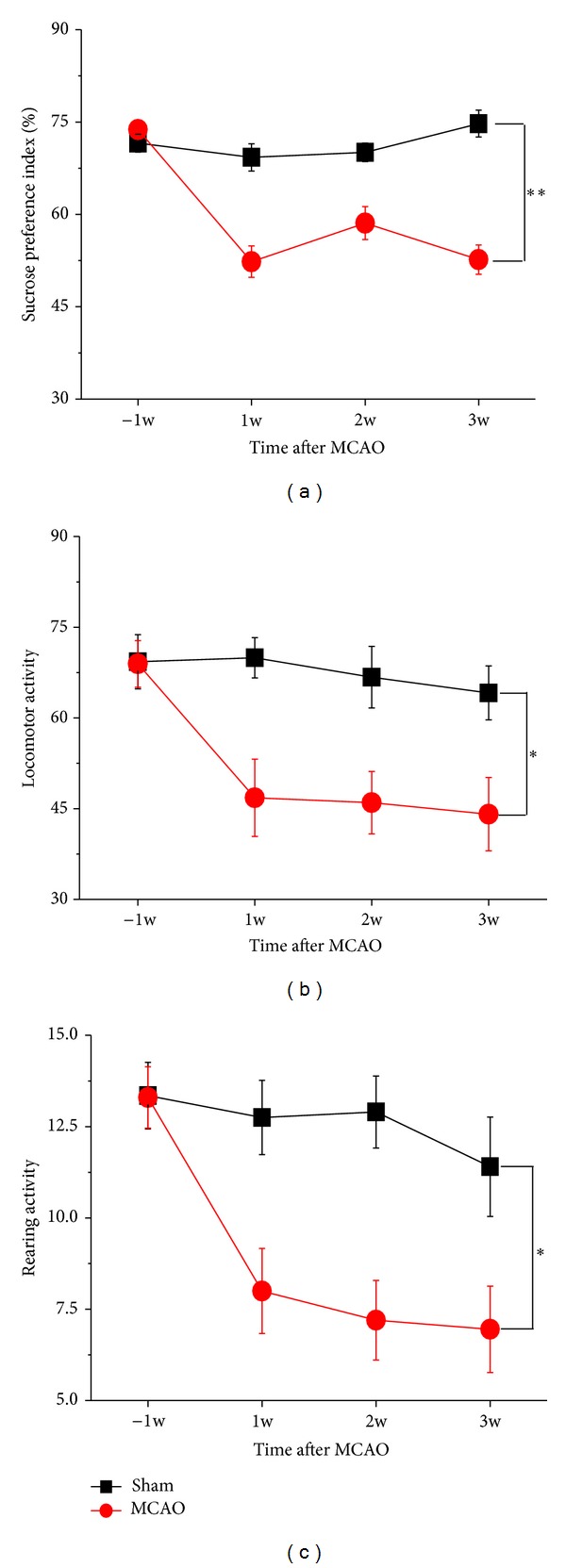
PSD is observed in OVX rats after focal ischemia (*n* = 20). (a) The percentage of sucrose intake was significantly decreased in OVX rats after MCAO, compared to the controls. Locomotor activity (b) and rearing activity (c) were also reduced in the MCAO animals, compared with sham-operated animals. Data were presented as mean ± SEM. *P < 0.05, **P < 0.001.

To determine whether focal cerebral ischemia could induce depressive-like behaviors, behavioral tests were performed in rat after focal ischemia. Compared with the sham-operated group, the ischemic rats displayed a reduction in sucrose preference (*F*(1,38) = 80.688, *P* < 0.001), which reached statistical significance at the first week and persisted at least over 3 weeks (Figure 1(a)). In addition, the poststroke rats also showed a reduction in locomotor activity (*F*(1,38) = 10.695, *P* < 0.05) and rearing activity (*F*(1,38) = 12.699, *P* < 0.05) at first week after MCAO, which continued to decline in the following sessions, compared with sham-operated animals, indicating that the depressive-like behaviors were developed at 1 week in poststroke rats (Figures 1(b) and 1(c)).

### 3.2. Administration of E_2_ Attenuated Poststroke Depressive-Like Behaviors

**Figure 2:**
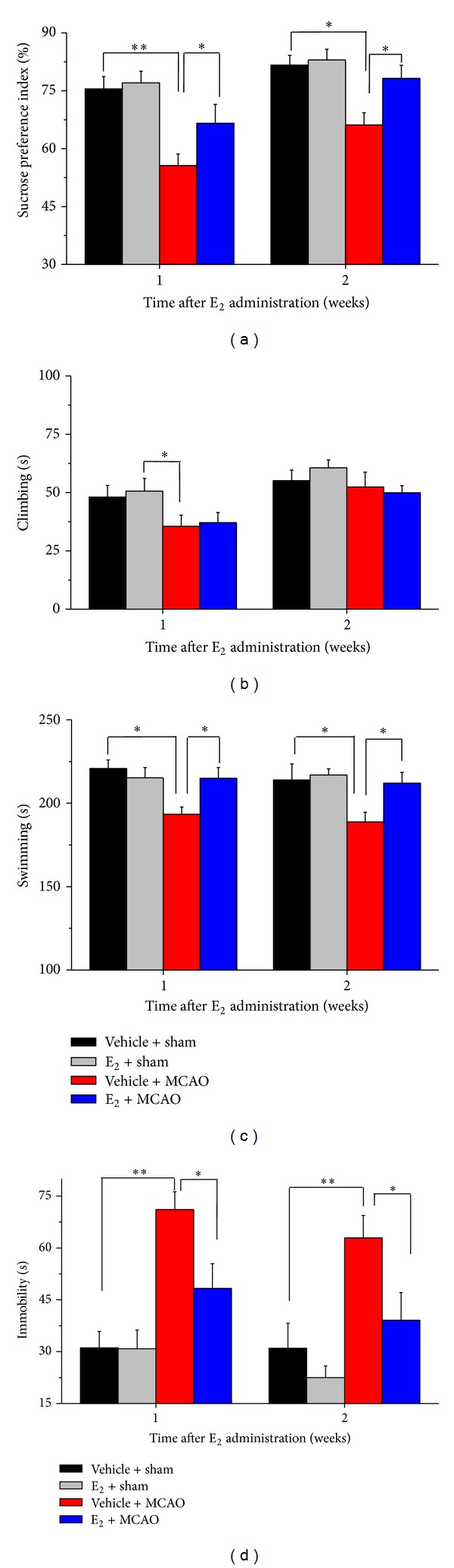
E_2_ administration after ischemic stroke reverses depressive-like behavior (*n* = 10). (a) E_2_-treated animals showed increased percentage of sucrose consumption at 1 week and 2 weeks after E_2_ administration, compared to vehicle-treated animals. (b) E_2_-treated animals showed no significant differences to vehicle-treated on climbing ability. E_2_-treated rats showed increased swimming behavior (c) and decreased immobility (d) after focal ischemia, compared to vehicle-treated ischemic animals. Data were presented as mean ± SEM. *P < 0.05, **P < 0.001.

To investigate whether E_2_ has effects on depressive-like behaviors in poststroke rats, we performed sucrose preference test and forced swimming test. In the sucrose preference test, we found that estradiol have increased sucrose preference index in E_2_ + MCAO group since the first week administration, compared to vehicle + MCAO group (1 W, *F*(3,36) = 7.715, 2 W, *F*(3,36) = 7.093, all *P < 0.05). In addition, sucrose preference indexes in both vehicle + sham group (1 W, *P* < 0.001; 2 W, *P* < 0.05) and E_2_ + sham group (1 W, 2 W, all **P < 0.001) were higher than the vehicle + MCAO group (Figure 2). In the forced swimming test, the longest immobility time was observed in vehicle + MCAO group, compared to the E_2_ + MCAO group (1 W, *F*(3,36) = 11.127, 2 W, *F*(3,36) = 7.177, all *P < 0.05), the vehicle + sham group (1 W, **P < 0.001, 2 W, *P < 0.05), and the E_2_ + sham (1 W, 2 W, all *P < 0.001) after E_2_ treatment for one week. However, these differences disappear at 2 weeks after E_2_ treatment, which was mainly due to an increase in swimming behavior (1 W, *F*(3,36) = 4.741, 2 W, *F*(3,36) = 3.664  *P < 0.05) (Figure 2).

### 3.3. E_2_ Treatment Did Not Affect Infarct Volumes after MCAO

**Figure 3:**
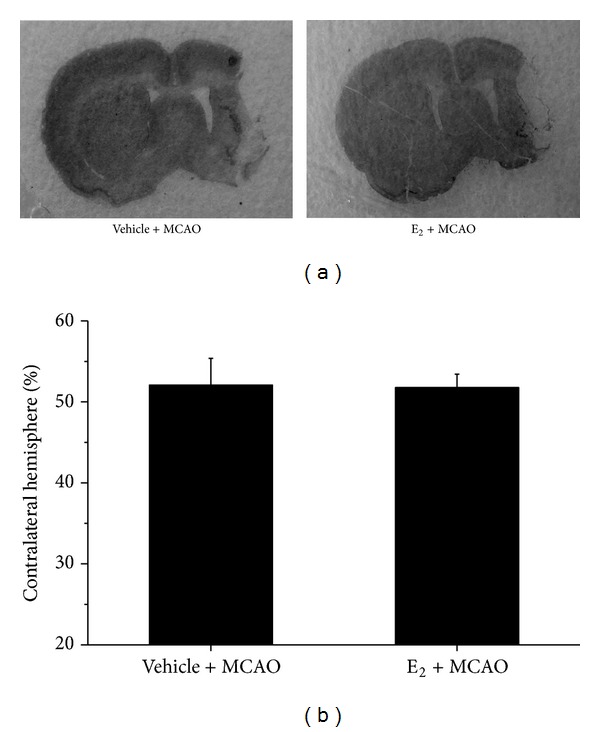
Infarct volume after MCAO with and without E_2_ treatment (*n* = 5). (a) Representative images of cresyl violet-stained coronal brain sections from vehicle- and E_2_-treated rats. (b) Quantification of infarct volumes in vehicle- and E_2_-treated rats. There were no significant differences between vehicle- and E_2_-treated groups.

To investigate whether the ischemic infarct volumes could be attenuated by E_2_ administration, rats were sacrificed 2 weeks after E_2_ administration, and the brains were removed and stained with cresyl violet. As shown in Figure 3, there was no significant reduction in infarction volume of E_2_-treated ischemic rats, compared with vehicle-treated group.

### 3.4. E_2_ Increased Neurogenesis after Ischemic Stroke

**Figure 4:**
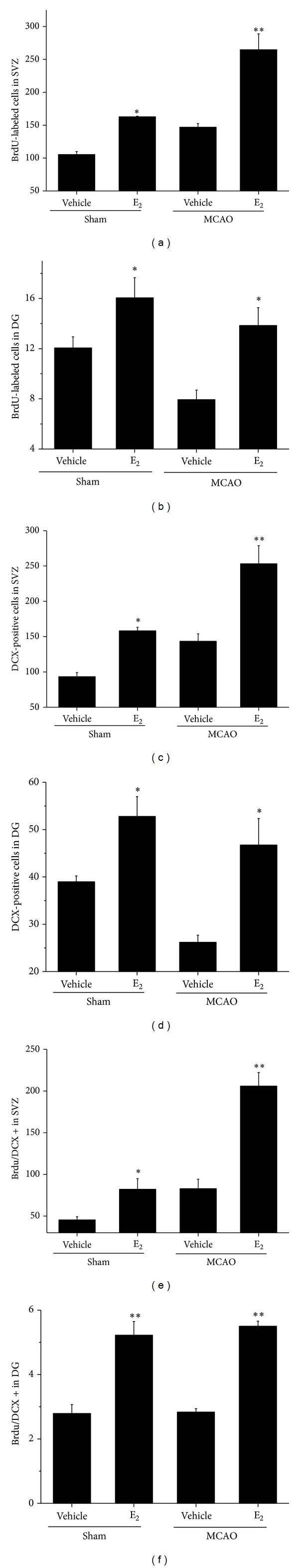
Effect of E_2_ on neurogenesis after focal ischemia (*n* = 4). ((a)-(b)) Quantification of BrdU-immunoreactive cells in the SVZ and the DG at the 5 weeks after sham-operated or MCAO rats treated with vehicle or E_2_. Data were presented as mean ± SEM. *P < 0.05; **P < 0.01 compared to vehicle. ((c)-(d)) Quantification of DCX-immunoreactive cells in SVZ and DG at the 5 weeks after sham-operated or MCAO rats treated with vehicle or E_2_. Data were presented as mean ± SEM. *P < 0.05; **P < 0.01 compared to vehicle-treated group. ((e)-(f)) Quantification of BrdU-immunoreactive cells in the SVZ and DG at the 5 weeks after sham-operated or MCAO rats treated with vehicle or E_2_. Data were presented as mean ± SEM. *P < 0.05; **P < 0.01 compared to vehicle.

**Figure 5:**
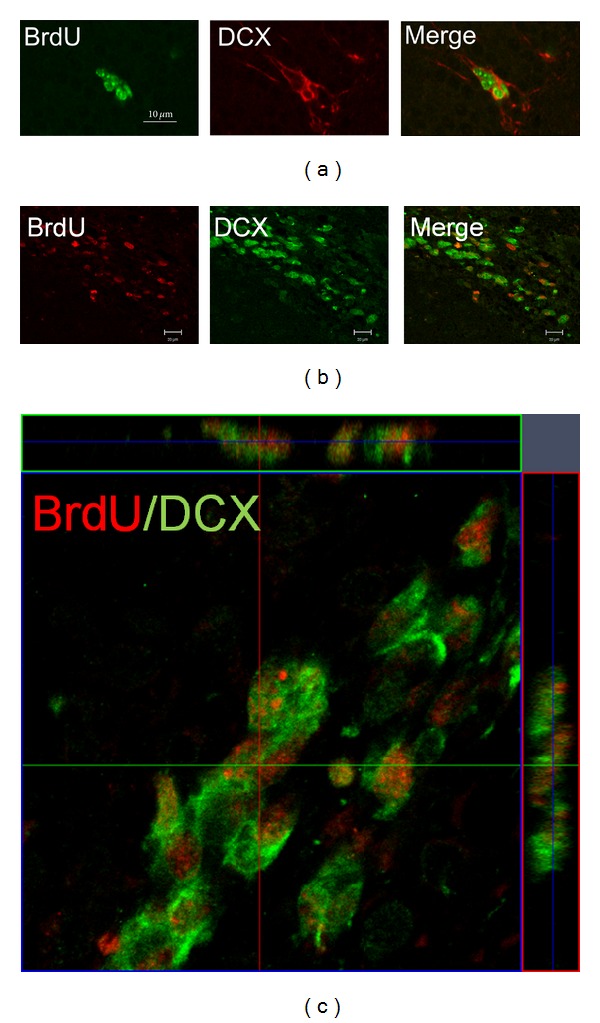
BrdU/DCX-positive cells in the DG and SVZ detected by confocal microscope. (a) Double-immunostaining shows that the BrdU (green) is co-localized with the DCX (red). Representative confocal images of BrdU/DCX-positive cells in the DG of hippocampus. (b) Representative confocal images of BrdU/DCX-positive cells in the SVZ in rat. (c) Confocal image stacks confirmed that BrdU-positive cells (red) expresses DCX (green) in the SVZ.

To determine whether E_2_ administration could enhance neurogenesis in the SVZ and DG of ischemic brain, rats were treated for 3 days with BrdU, which labels cells that undergo DNA replication in S-phase and therefore reflects the current rate of cell division. As shown in Figure 4, BrdU- and DCX-positive cells in the SVZ and DG were significantly increased in E_2_-treated rats compared with control animals (*P < 0.05). An increase of BrdU- and DCX-positive cells in the SVZ was also observed in E_2_ + MCAO group, compared to vehicle + sham group. Interestingly, BrdU- and DCX-positive cells in the DG were decreased after focal ischemia, compared to the sham-operated rats (*P < 0.05), which was reversed after E_2_ administration (*P < 0.05). Confocal images show that BrdU-positive cells expressed DCX, suggesting that these BrdU-positive cells were proliferative neuronal progenitor cells, and double-labeled cells in E_2_-treated group were significantly increased compared to vehicle-treated group after ischemia (**P < 0.001) (Figure 5).

## 4. Discussion

In the present study, we developed a rat model of PSD using left MCAO and found that E_2_-treated PSD rats showed significant improvement in their behavioral performance, as measured by the sucrose preference test and the forced swimming test, suggesting that administration of E_2_ induces antidepressant like effect in PSD rats. In addition, our results also showed that the E_2_-mediated depressive-like behavioral improvements were concomitant with a significantly increased neurogenesis in the DG after focal ischemia, suggesting that neurogenesis may play a critical role in E_2_-mediated antidepressant effect after focal ischemia.

Clinical evidence show that the susceptibility to develop depression in women increases when the estrogen levels fluctuate during their life [27, 28]. Estrogen replacement therapy in these women may reduce depressive symptoms during the premenopausal and postpartum periods [29]. E_2_ also induces antidepressant like effects in animal models of depression [30–32]. Furthermore, it has been suggested that E_2_ can enhance and shorten the antidepressant-like action of various antidepressants, when combined with these antidepressants [33, 34]. However, whether E_2_ produces antidepressant effect in animal model of poststroke depression remains unknown. Here, we applied two behavioral tests, including the sucrose preference test and the force swimming test, which have been developed as straightforward tests for screening the efficacy of antidepressants, to investigate whether E_2_ induces antidepressant effect. We observed that E_2_ treatment reversed depressive-like behavior in PSD rats, by increasing sucrose consumption in the sucrose preference test, decreasing the immobility time and increasing swimming time in the force swim test, which is consistent with the suggested antidepressant-like effect of E_2_.

Previous studies have shown that neurogenesis plays a critical role in the antidepressant-mediated behavioral effects [19]. Therefore, we hypothesized that the antidepressant effects produced by E_2_ may also correlate with increased neurogenesis. Our data in this study supported this hypothesis. After 2 weeks of E_2_ treatment, the increased neurogenesis in the DG and SVZ was observed by immunohistochemistry analysis. Increased BrdU/DCX-expressing cells in the DG and SVZ suggest that newborn neurons were generated in these regions. Our results are consistent with a recent finding that E_2_ treatment enhanced neurogenesis in the SVZ and DG and improved behavioral recovery after ischemic stroke [12]. Suzuki and his coworker also found that pretreatment with physiological doses of E_2_ promoted neurogenesis in the SVZ in female rats after ischemic stroke [35]. McClure and her colleagues showed that rats injected with E_2_ showed significantly higher levels of activation of new neurons in response to spatial memory compared to controls, suggesting that E_2_ plays a role in activation of new neurons in the hippocampus in response to spatial memory in adult female rats [36]. The fate of stem cells in the SVZ remains unclear; some may migrate into the olfactory bulb (OB) via the rostral migratory stream (RMS) and differentiate, where upon they differentiate into local interneurons. As focal ischemia damages cortex and striatum, but does not affect hippocampus neurons, the new born cells migrate into the damaged regions and differentiate into functional neurons for repair. The newborn cells in the SGZ migrate into the granular layer of DG and differentiate into mature neurons. Growing evidence shows that hippocampal adult neurogenesis is important for learning and memory. Most likely, E_2_ induced neurogenesis in the SGZ mainly contributes PSD recovery.

Although our data did not provide direct evidence to show that the effects of E_2_, on neurogenesis and antidepression, are linked, the role of neurogenesis in E_2_-mediated antidepressant effects is further suggested by the following observations: (1) depletion of neurogenesis in the mouse hippocampus by X-irradiation blocks antidepressant effects of two classes of antidepressants [19]; (2) chronic cannabinoid HU210 treatment produces antidepressant effects that depend on hippocampal neurogenesis [37]; (3) the antidepressant effect induced by fluoxetine also requires increased neurogenesis [38]. The underlying mechanism by which E_2_ acts to increase neurogenesis remains unclear, but likely involves participation of estrogen receptors and neurotrophic factors. Both estrogen receptor *α* (ER*α*) and estrogen receptor *β* (ER*β*) have been detected in the DG of hippocampus in the rat, suggesting that these receptors could influence estradiol's effects on neurogenesis [39, 40] as knocking out either of these receptors blocks the ability of estradiol to increase neurogenesis, indicating that both receptors could directly mediate estradiol's effects on neurogenesis [12, 35]. One of candidate growth factors involved in estradiol's neurogenic effect is brain-derived neurotrophic factor (BDNF), as studies have shown that estrogens regulate the BDNF expression [41, 42] and promote the survival of young granule neurons by stimulating expression of BDNF and its receptor in the hippocampus [43]. Notably, our neurogenesis data shown here are different from other reports [44], the possible reasons include the different animal species, different stroke models, different duration and time to inject BrdU, and whether ovariectomy surgery was performed. Interestingly, a recent study shows that FST increased immobility and corticosterone levels in OVX but not in rats in proestrus. In addition, FST did not affect cell proliferation but significantly decreased the number of BrdU-labeled cells at 2 hr only in OVX-rats, an effect that remained for 3 and 14 days after FST, suggesting that acute stress further decreases the effect of ovariectomy on immobility behavior and hippocampal cell survival in rats [45].

Taken together, all these lines of evidence support the notion that increased neurogenesis in DG and SVZ appears to underlie the mechanism of antidepressant effects produced by E_2_ treatment in poststroke depression rats. However, whether increased neurogenesis is sufficient to produce the entire antidepressant effects of E_2_ remains to be addressed.
